# Impact and cost-effectiveness of chlamydia testing in Scotland: a mathematical modelling study

**DOI:** 10.1186/1742-4682-12-2

**Published:** 2015-01-15

**Authors:** Katharine J Looker, Lesley A Wallace, Katherine ME Turner

**Affiliations:** School of Social and Community Medicine, University of Bristol, Canynge Hall, 39 Whatley Road, Bristol, BS8 2PS UK; NHS National Services Scotland, Glasgow, UK

**Keywords:** Chlamydia, Sexually transmitted infections, Mathematical modelling, Epidemiology, Cost-effectiveness, Scotland, Young people, Pelvic inflammatory disease, Tubal factor infertility

## Abstract

**Background:**

Chlamydia is the most common sexually transmitted bacterial infection in Scotland, and is associated with potentially serious reproductive outcomes, including pelvic inflammatory disease (PID) and tubal factor infertility (TFI) in women. Chlamydia testing in Scotland is currently targeted towards symptomatic individuals, individuals at high risk of existing undetected infection, and young people. The cost-effectiveness of testing and treatment to prevent PID and TFI in Scotland is uncertain.

**Methods:**

A compartmental deterministic dynamic model of chlamydia infection in 15–24 year olds in Scotland was developed. The model was used to estimate the impact of a change in testing strategy from baseline (16.8% overall testing coverage; 0.4 partners notified and tested/treated per treated positive index) on PID and TFI cases. Cost-effectiveness calculations informed by best-available estimates of the quality-adjusted life years (QALYs) lost due to PID and TFI were also performed.

**Results:**

Increasing overall testing coverage by 50% from baseline to 25.2% is estimated to result in 21% fewer cases in young women each year (PID: 703 fewer; TFI: 88 fewer). A 50% decrease to 8.4% would result in 20% more PID (669 additional) and TFI (84 additional) cases occurring annually. The cost per QALY gained of current testing activities compared to no testing is £40,034, which is above the £20,000-£30,000 cost-effectiveness threshold. However, calculations are hampered by lack of reliable data. Any increase in partner notification from baseline would be cost-effective (incremental cost per QALY gained for a partner notification efficacy of 1 compared to baseline: £5,119), and would increase the cost-effectiveness of current testing strategy compared to no testing, with threshold cost-effectiveness reached at a partner notification efficacy of 1.5. However, there is uncertainty in the extent to which partner notification is currently done, and hence the amount by which it could potentially be increased.

**Conclusions:**

Current chlamydia testing strategy in Scotland is not cost-effective under the conservative model assumptions applied. However, with better data enabling some of these assumptions to be relaxed, current coverage could be cost-effective. Meanwhile, increasing partner notification efficacy on its own would be a cost-effective way of preventing PID and TFI from current strategy.

**Electronic supplementary material:**

The online version of this article (doi:10.1186/1742-4682-12-2) contains supplementary material, which is available to authorized users.

## Introduction

Chlamydia is the most common sexually transmitted bacterial infection in Scotland, as in the rest of Europe [1, 2]. In 2011 there were 18,961 diagnoses of genital chlamydia infection in Scotland: 11,881 in women and 6,913 in men (and 167 of unknown gender) [3]. Diagnoses are highest among young people [3]. In 2010, a quarter of young women (25.6%) and 8.3% of young men aged 15–24 years were tested for chlamydia (16.8% average coverage between women and men) [4, 5]. Of these, 10.6% of females and 15.4% of males were positive for chlamydia infection: an average positivity of 11.8% [3, 4]. Chlamydia infection is often asymptomatic [6, 7], so the number of diagnoses is an underestimate of the total number infected. In the third National Survey of Sexual Attitudes and Lifestyles (Natsal-3), 5.7% (95% C.I. 2.2, 14.2) of men and 3.1% (95% C.I. 1.1, 8.6) of women aged 16–24 in Scotland were infected with chlamydia: 4.4% prevalence when averaged between the sexes [8] (Scottish-specific prevalence data kindly provided by Natsal-3 researchers). Infection is curable with antibiotics, and antibiotic treatment is a standard part of the care of chlamydia-positive individuals. Untreated infection spontaneously clears in the majority of individuals within some months (1.13-1.63 years in women [9, 10]), but infection can persist for years [11]. After clearance (spontaneous or treatment-induced) individuals remain at risk of reinfection from untreated infected partners or new (infected) partners, while “treated” individuals may not clear their first infection due to treatment failure [12, 13].

Chlamydia infection is associated with pelvic inflammatory disease (PID), tubal factor infertility (TFI) and ectopic pregnancy (EP) [14]. PID is often assumed to be a necessary intermediate stage between chlamydia infection and TFI or EP, though a proportion of PID may be subclinical. Estimation of the risk of progression to secondary outcomes through observational studies and randomised controlled trials is difficult for a number of reasons, including: (1) the outcomes are rare; (2) the outcomes may occur many years after chlamydia infection; (3) outcomes may not always be diagnosed; (4) treatment may not prevent all outcomes; (5) outcomes have multiple causes besides chlamydia; and (6) the risk of complications may vary with duration or number of infections [15, 16]. Recently statistical modelling approaches have been applied to better quantify the risks of long-term outcomes associated with chlamydia, based on observed data, but with adjustment for inherent weaknesses of observational studies [17].

Most European guidelines for chlamydia management include treatment, partner notification and health promotion, and in some instances, recommendation of retesting of positives after three months [18–20]. National screening programmes exist in some European countries (England: opportunistic screening programme; the Netherlands: population register screening) while others have high levels of opportunistic testing in the absence of an organised national programme (Denmark, Estonia, Iceland, Latvia, Norway and Sweden). In Scotland, the 2009 Scottish Intercollegiate Guidelines Network (SIGN) guidelines state that chlamydia testing should be focussed on symptomatic patients and asymptomatic individuals at high risk of having an undiagnosed infection, and targeted to young people [21]. There is no current recommendation for opportunistic testing of all young people.

Robust estimates of the impact of testing programmes and their cost-effectiveness are hampered by uncertainties in the risks of disease progression, quality-adjusted life years (QALYs) applied to chlamydia and its sequelae [22], and the effectiveness of testing and treatment to reduce chlamydia prevalence and prevent progression at a population level. Transmission dynamic mathematical models are an appropriate tool to investigate the potential impact and cost-effectiveness of different testing strategies, and to explore the effect of uncertainties in the underlying parameter assumptions. Here we apply the latest estimates of progression, the effect of testing and treatment, and QALY loss associated with progression, to a deterministic model to evaluate the impact and cost-effectiveness of current and potential testing strategies to prevent PID and TFI in Scotland.

## Methods

We developed a deterministic, compartmental dynamic model of chlamydia transmission and outcomes in the Scottish population aged 15–24 years. We used this to evaluate the impact and cost-effectiveness of current and potential testing strategies (which correspondingly also include treatment) to prevent PID and TFI in Scotland. We considered current chlamydia testing coverage and partner notification rates, and potential alternative strategies. We estimated the total cost of chlamydia testing (including cost of outcomes), the number of PID and TFI cases prevented, and the cost per outcome prevented.

### Model structure

**Figure 1:**
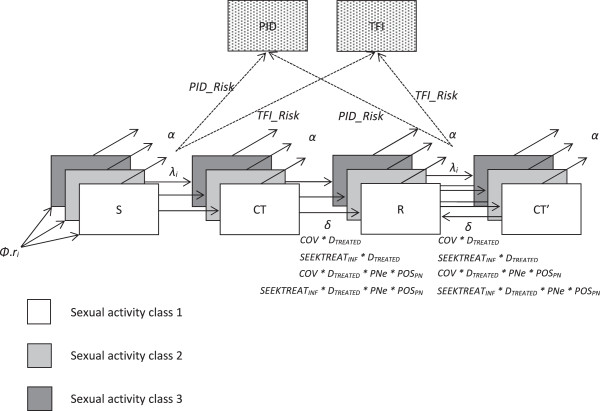
**Model structure.** Individuals enter the model on their 15^th^ birthday and exit on their 25^th^ birthday. All individuals entering the population are initially susceptible (S) to chlamydia infection, and enter each sexual activity class *i* (defined by partner contact rate *c*_*i*_) in proportions corresponding to the fraction of the population in each class (*r*_*i*_). The flows represent ageing into and out of the model (*φ* and *α*), and chlamydia infection (into CT), recovery (into R) and reinfection (into CT’). Infection rates for each sexual activity class are given by the force of infection, *λ*_*i*_. In addition to natural recovery (*δ*), a proportion of individuals recover by seeking treatment (*SEEKTREAT*_*INF*_ * *D*_*TREATED*_), while further fractions recover through additional testing and treatment (*COV* * *D*_*TREATED*_), or by partner notification and treatment (*PNe* * *POS*_*PN*_) applied to treated index cases. A proportion of females (half of the model population) with incident infection develops PID, while a smaller proportion develops TFI. For a full explanation of the model structure see Methods text.

A compartmental deterministic model of the heterosexual transmission of chlamydia in Scotland was developed and solved numerically (Runge–Kutta 4; dt = 0.01) in Berkeley Madonna, illustrated in Figure 1. The model population was stratified by sexual activity class *i* but not sex, with outputs then scaled to the numbers of females and males aged 15–24 years in Scotland, *N*_*TARGET_F*_ and *N*_*TARGET_M*_. The reason for this approach is that incorporation of sex would have necessitated complex model fitting to fit to the unequal observed chlamydia prevalence, testing coverage and test positivity between women and men. Most likely this would have necessitated making additional assumptions for the pattern of sexual mixing between different sexual activity classes by sex, the way in which the numbers of partnerships between the sexes are balanced, treatment seeking behaviour between the sexes, and the transmission probabilities for women and men, and hence, introducing even greater uncertainty in the model. Moreover, the interpretation and resultant policy implications of a model fitted to unequal testing coverage and unequal test positivity between the sexes, are unclear.

Individuals enter the model at age 15 susceptible to chlamydia infection, and leave at age 25. Entry rate *φ* and ageing rate *α* were selected to maintain a constant population. The proportion of the population in each sexual activity class, *r*_*i*_, and the partner change rates per year for each class, *c*_*i*_, were based on the numbers of partners in the past year reported in the second National Survey of Sexual Attitudes and Lifestyles (Natsal-2) [23]. All modelled individuals (i.e., aged 15–24 years) can potentially form partnerships with all other modelled individuals: there is no further stratification of the model by age. We assumed slight assortative mixing, *ϵ*, between sexual activity classes. This means that there is some tendency for individuals to form partnerships with other individuals in the same sexual activity class as themselves, over and above the proportion of partnerships that would be expected based on the average rates of partnership formation of each of the different sexual activity classes.

Individuals are infected with chlamydia according to the per-susceptible force of infection *λ*_*i*_. Recovered individuals remain equally susceptible to infection (Susceptible-Infected-Susceptible (SIS) model). A proportion of females with incident chlamydia infection progresses to PID at rate *PID_risk*, and a smaller proportion progresses to TFI at rate *TFI_risk*. PID and TFI cases are counted for the age at incident chlamydia infection (15–24 years), although in reality there may be a substantial delay between infection and the outcome. Due to uncertainties in the natural history of chlamydia outcomes and the effect of treatment, we conservatively assume that the outcome risks are unaffected by treatment for chlamydia, and that the risks are the same whether the incident infection is a first infection or a subsequent infection.

Once infected with chlamydia, individuals recover spontaneously from infection on average after one year (rate *δ*). Otherwise, individuals may recover either: (1) by actively seeking testing and treatment; (2) through additional testing and treatment; or (3) via partner notification and treatment. Specifically, we apply a rate of active treatment seeking (*SEEKTREAT*_*INF*_) to those assumed to be at high risk of infection (e.g., symptomatic individuals, or individuals engaging in high-risk behaviour). The positivity in this group (*POS*_*SEEKTREAT*_) is higher than the population prevalence, but slightly lower than the prevalence among partners (*POS*_*PN*_), as this was considered most plausible. We also apply a rate of additional testing to all individuals who would not otherwise get tested (*COV*). Here we assume that the proportion infected is equal to the population prevalence (*PREV*). Lastly, we apply a rate of partner notification (*PNe*) to treated positive (index) cases identified through either additional testing or treatment seeking. It is assumed that there is some loss-to-follow-up (1 - *D*_*TREATED*_) between testing and treatment for positive cases identified by additional testing or through treatment seeking, but not for partner notification (i.e., treatment is given at the same time as partner testing). This assumption was informed by discussion with Scottish genitourinary medicine clinicians.

Full model equations are given in the Additional file 1, along with the equations for the model outputs (Additional file 1: Table S1). The model parameters are detailed in Table 1. Of note, there is uncertainty in a number of the model parameters (Table 1). As a consequence of this, and to enable ease of model fitting and subsequent interpretation, we did not introduce further complexity in the model for which parameterisation would be potentially even more problematic/uncertain: for example, symptomatic vs asymptomatic chlamydia infection, chlamydia immunity or condom use, although for condom use, the transmission probability will be an average across all partnerships and will be the effective transmission probability, and thus will implicitly include average condom use.

**Table 1:** **Model parameters**

Parameter	Symbol	Baseline value	Range	Source	Notes
**Chlamydia natural history**					
Transmission probability per partnership	*β*	0.346 per partner	--	Calibrated by model fitting	Estimated by fitting model predictions to chlamydia prevalence and overall testing coverage. Modelled by fixing duration of infection and allowing transmission probability to vary – method also used by Althaus [24] and Clarke [25]
Rate of recovery from infection per year	*δ*	1 per yr	--	[9, 10]	No estimates in men; likely shorter than in women. Estimates in women from recent modelling studies: 14 months [9] and 16 months [10]. Assumed shorter duration overall to include men. Model transmission modified to fit to desired prevalence, and transmission probability and duration correlated
Risk of PID in those with incident chlamydia	*PID_risk*	0.16	0.06-0.25	[17]	Range derived from literature estimates
Risk of TFI in those with incident chlamydia	*TFI_risk*	0.02	0.01-0.04	[26]	Range derived from literature estimates
**Demography**					
Female population in Scotland aged 15–24 years	*N*_*TARGET_F*_	335,518	--	[5]	Population estimate as at 30 June 2010
Male population in Scotland aged 15–24 years	*N*_*TARGET_M*_	349,417	--	[5]	Population estimate as at 30 June 2010
Rate of entry into the model per year	*φ*	1/10 per yr	--		
Rate of ageing from model per year	*α*	1/10 per yr	--		
**Sexual behaviour**					
Proportion recruited into activity group *i*	*r*_*i*_	*r*[1] = 0.702; *r*[2] = 0.230; *r*[3] = 0.068	--	[23]	
Partner contact rate per year in those in activity group *i*	*c*_*i*_	c[1] = 0.674; c[2] = 2.538; c[3] = 9.452	--	[23]	
Mixing between sexual activity classes	*ϵ*	0.2	--	[27]	Based on previous estimates where 0 represents proportionate mixing and 1 fully assortative mixing
**Testing and treatment**					
Baseline prevalence among females and males aged 15–24 years	*PREV*	4.4%	--	[8]	Based on prevalence among 16–24 year olds in Scotland (Scottish-specific prevalence data kindly provided by Natsal-3 researchers)
Overall testing coverage	*TEST*	16.8%	8.4%, 16.8%, 25.2%, 33.6%, 42.0	Stepwise values (0.5, 1, 1.5, 2, 2.5 increases relative to baseline) across an assumed realistic range	Note: the overall testing coverage includes all types of test (additional testing, treatment seeking and partner notification) and is the coverage at baseline partner notification efficacy (0.4). Changes in partner notification result in small changes in overall coverage but which are not shown on the figures for simplicity
Additional testing coverage	*COV*	11.9%	2.3%, 11.9%, 21.5%, 31.0%, 40.5%	Calibrated by model fitting	Estimated from fitting model predictions to chlamydia prevalence and overall testing coverage
Percentage of additionally tested individuals, or individuals seeking treatment (females or males) identified as positive who are successfully treated	*D*_*TREATED*_	91%	--	NCSP 2011-2012 [28]	NCSP target is 95%. It is assumed that treatment is only given after a positive test result, and that there can therefore be loss to follow-up between testing and treatment. This does not include treatment failure, which is not incorporated in the model
**Treatment seeking behaviour**					
Proportion of all those infected who seek treatment	*SEEKTREAT*_*INF*_	0.2	--	[24]	Selected for convenience to differentiate treatment seeking behaviour which is not dependent on policy i.e., based on symptoms or contact with infected partner, and testing of asymptomatic individuals that could be modified depending on testing strategy adopted. Althaus *et al.* found that the value of the proportion symptomatic and seeking treatment a short time after infection did not have a substantial effect on transmission dynamics
Proportion of all those seeking treatment who are infected with chlamydia	*POS*_*SEEKTREAT*_	0.2	--	Assumed realistic value	Chosen to be slightly lower than assumed prevalence among partners
**Partner notification**					
Number of partners successfully notified and tested/treated per treated index (from either additional testing or treatment seeking testing) (=partner notification efficacy)	*PNe*	0.4	0.0-2.0 in 0.25 increments	[29]	Range within the number of partners reported by index cases (e.g., NCSP range 0.1-1 partner notified per index)
Percentage positive among partners tested	*POS*_*PN*_	30%	--	[30]	
**Costs**					
Cost of a test, including treatment for those positive (average cost)	*C*_*TEST*_	£45	Percentage change: -50% to +100%	[31]	Does not vary with population prevalence. NAO says it should be possible to do a test for £33 [32]
Cost of partner notification per partner, including testing and treatment for those positive (average cost)	*C*_*PN*_	£114	Percentage change: -50% to +100%	[31]	Does not vary with population prevalence
Cost of treating PID	*C*_*PID*_	£163	--	[33]	
Cost of treating TFI	*C*_*TFI*_	£2,115	--	[34]	Cost of one round of IVF on the NHS (conservatively costed in order to account for those infertile women who do not undergo IVF)
**Health state utility**					
PID	--	0.9	Percentage change: -50% to +100%	[22]	Applies for 3 months
TFI	--	0.76	Percentage change: -50% to +100%	[22]	Applies for 1 year

### Model fitting

The chlamydia transmission probability per partnership, *β*, and the additional testing coverage at baseline, *COV*, were calibrated by fitting the model at equilibrium to 4.4% average chlamydia prevalence between women and men (*PREV*) and to 16.8% average overall testing coverage between women and men (i.e., all types of testing) (*TEST*), using the curve fitting function in Berkeley Madonna. We assumed a baseline of 0.4 partners notified and tested/treated per treated positive index. This gave a transmission probability of 0.346 per partnership, and 11.9% additional testing coverage.

### Analysis

We explored the impact of current and alternative strategies by varying both overall testing coverage (*TEST*; recalibrating *COV*), and partner notification efficacy (*PNe*). Stepwise values for the alternative overall testing coverage across a range we considered plausible were obtained by multiplying the overall testing coverage at baseline (16.8%) by 0.5, 1, 1.5, 2 and 2.5. The outcomes were the change in the number of PID and TFI cases prevented per year from a baseline overall testing coverage of 16.8% and 0.4 partner notification efficacy, and the expenditure on testing and treatment per PID case or TFI case prevented compared to no chlamydia testing (additional testing or treatment seeking) or partner notification. The comparison for cost (and subsequent overall cost-effectiveness calculations) was no testing in order to evaluate the total cost of testing and treatment for all cases prevented, and not because no testing is a realistic management strategy.

In our model, the full effect of changing coverage and/or partner notification activities is not realised until some time after the change is implemented. However, the time taken is predicted to be reasonably quick (the time taken is dependent on the scale of the change, but generally speaking most of the impact is realised within five years). In practice, “real-world” factors such as speed of policy implementation, and other factors such as age-dependent sexual mixing, will likely more strongly influence time taken to reach full impact of changing testing strategy. For this reason, and to enable direct comparison between strategies, it was decided to present all analyses of change in testing strategy for the equilibrium state, i.e., time-independent.

Sensitivity analyses were performed for the change in the number of outcomes prevented and the expenditure on testing and treatment per case averted, varying the assumed PID and TFI risks. We selected a value for the overall testing coverage of 25.2% (a 50% increase in overall coverage compared to baseline) for these analyses since this was in the middle of the modelled range of overall coverages.

### Cost-effectiveness calculation

We selected health state utility values of 0.9 for PID [35] (which applies for 3 months), and 0.76 for TFI [35] (which applies for 1 year), from the values presented in Jackson *et al.*[22]. The health state utility value for TFI may be lower than 0.76 for 1 year for women who have not been able to get pregnant and/or who have endured several cycles of IVF; however, this value needs to be an average across all women with TFI, some of whom may never attempt to become pregnant and therefore remain unaware they are infertile. We adjusted the value of 0.9 for 3 months for PID to apply for a year: (0.25 * 0.90) + (0.75 * 1) = 0.975. The QALY estimate for each outcome is given by (1 - heath state utility value). We calculated the cost of chlamydia testing per quality-adjusted life year (QALY) gained:


Sensitivity analyses were performed for the cost per QALY gained at 16.8% coverage compared to no testing: (1) varying chlamydia testing costs; (2) varying the number of QALYs gained by averting PID and TFI; and (3) varying the effectiveness of treating chlamydia to prevent PID and TFI. An overall testing coverage of 16.8% (i.e., current overall coverage) was selected since the cost-effectiveness of current testing strategy is unknown and therefore it was considered that assessing the sensitivity of cost-effectiveness calculations at this level would be of greatest interest.

## Results

### Impact of chlamydia testing

In Scotland the average chlamydia testing coverage among 15–24 year olds is 16.8% and the average chlamydia prevalence is 4.4% in this age group. The number of partners notified and tested/treated per treated index is assumed to be 0.4. These values form the baseline strategy (current testing situation) for comparison with alternative testing strategies.

The modelled test positivity at baseline testing strategy is 9.3%, which is similar to the average observed positivity among females and males (11.8%). In Scotland, we estimate from our model that 3,366 cases of PID and 421 cases of TFI in women currently occur annually which are attributable to a chlamydia infection aged 15–24 years old. Under current testing strategy we estimate that a further 2,062 cases of PID and 258 cases of TFI are prevented from occurring every year. The total annual cost of testing and treatment at this level is estimated from the model to be about £5.4 million.

In the model, increasing testing coverage by 50% from baseline to 25.2% results in *fewer* outcomes per year (703 fewer PID, 88 fewer TFI: a 21% decrease in the number of cases occurring, or 34% increase in the number of cases prevented, compared to baseline) (Additional file 1: Figures S1a and S1b). If the overall testing coverage is reduced by 50% from current levels to 8.4%, there will be an *increase* in outcomes (669 additional PID, 84 additional TFI: a 20% increase in the number of cases occurring, or 32% decrease in the number of cases prevented, compared to baseline) (Additional file 1: Figures S1a and S1b). Increasing partner notification results in fewer cases of PID and TFI. For example, an increase from 0.4 to 1 partners notified and tested/treated per positive treated index (16.8% overall coverage) results in an additional 372 and 47 PID and TFI cases prevented each year, respectively (Additional file 1: Figures S1a and S1b).

The model also shows that increasing partner notification always reduces the expenditure on testing and partner notification per case (PID or TFI) averted since partner positivity is higher than the positivity in index tests (treatment seeking and additional testing) (Additional file 1: Figures S2a and S2b). Increasing the overall testing coverage generally leads to increased expenditure per case (PID or TFI) averted, due to additional testing finding proportionately fewer infections than treatment seeking behaviour (Additional file 1: Figures S2a and S2b). If partner notification is high, then increasing additional testing can sometimes result in a reduction in the cost per case prevented.

These results are sensitive to the assumed PID and TFI risks (Additional file 1: Figures S3a and S3b). With higher PID and TFI risks, the number of cases prevented rises and the expenditure per case averted decreases relative to the default risks, and vice versa for lower risks. However, qualitative trends as a function of changing testing strategy are unaffected.

### Cost-effectiveness of chlamydia testing and treatment to prevent PID and TFI

**Figure 2:**
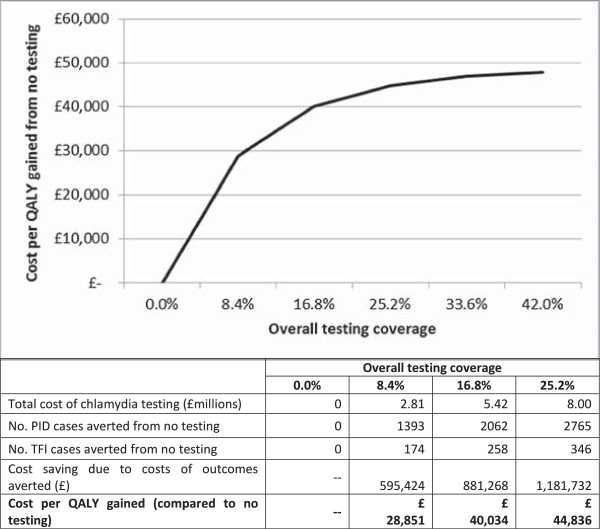
**Cost per QALY gained for different levels of overall testing coverage compared to no testing.** Partner notification efficacy is 0.4. Note that there is no valid cost per QALY gained for no testing. Calculations are for equilibrium state. For full table accompanying this Figure see Additional file 1: Table S2.

At current chlamydia testing levels of 16.8% overall testing coverage and 0.4 partners notified and tested/treated per treated index, the cost per QALY gained for PID and TFI compared to no testing is £40,034 (Figure 2 and Additional file 1: Table S2). This is above the threshold cost-effectiveness of £20,000-£30,000 used by the National Institute for Health and Care Excellence (NICE) [36]. The cost per QALY gained increases with increased testing coverage, and decreases with decreased testing coverage. This is because increasing testing coverage means increasing the number of additional tests among young people generally: all those seeking treatment (who have higher test positivity) are already being tested. At 8.4% overall testing coverage (50% of current coverage), the majority of tests are done in individuals seeking treatment and their partners, and the cost per QALY gained for PID and TFI compared to no testing is £28,851, which is at threshold cost-effectiveness. However, it should be remembered that more PID and TFI cases occur at lower testing coverage.

**Figure 3:**
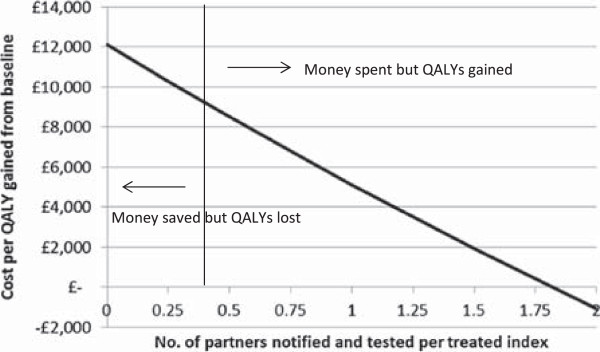
**Incremental cost per QALY gained if partner notification efficacy is changed from baseline (0.4).** Overall testing coverage is 16.8%. Note that there is no valid cost per QALY gained for the baseline strategy (0.4 partners notified and tested/treated per treated positive index; indicated by the vertical line on the figure). Calculations are for equilibrium state. For table accompanying this Figure see Additional file 1: Table S3.

Increasing partner notification efficacy on its own under current overall testing coverage is a cost-effective way of preventing PID and TFI. For example, an increase from 0.4 to 1 results in an incremental cost per QALY gained of £5,119 compared to baseline (16.8% overall testing coverage) (Figure 3 and Additional file 1: Table S3). Furthermore, at overall testing coverage of 16.8% and a partner notification efficacy of 1, the overall cost per QALY gained compared to no testing (i.e., taking all prevented cases into account) is £34,694, which is close to threshold cost-effectiveness (Additional file 1: Table S3). Threshold cost-effectiveness for current testing coverage is reached at a partner notification efficacy of 1.5. Thus, increasing partner notification activities would increase the cost-effectiveness of current overall testing coverage.

**Figure 4:**
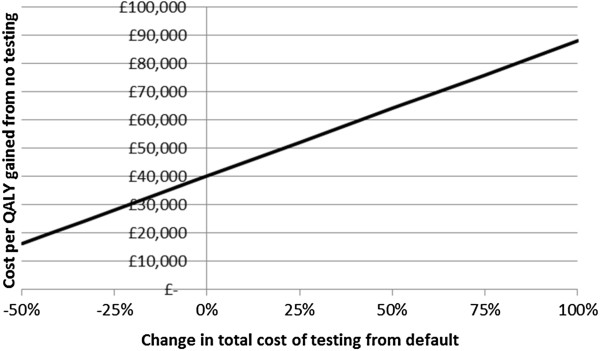
**Sensitivity analysis for the cost per QALY gained for baseline testing, varying the testing costs.** Baseline testing strategy is 16.8% overall testing coverage and 0.4 partner notification efficacy. The cost per QALY gained is compared to no testing.

**Figure 5:**
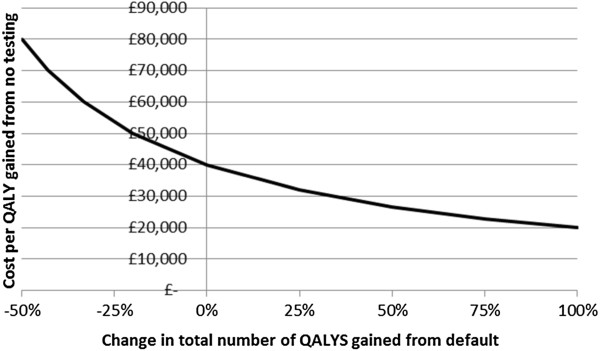
**Sensitivity analysis for the cost per QALY gained for baseline testing, varying the QALY gain.** Baseline testing strategy is 16.8% overall testing coverage and 0.4 partner notification efficacy. The cost per QALY gained is compared to no testing.

**Figure 6:**
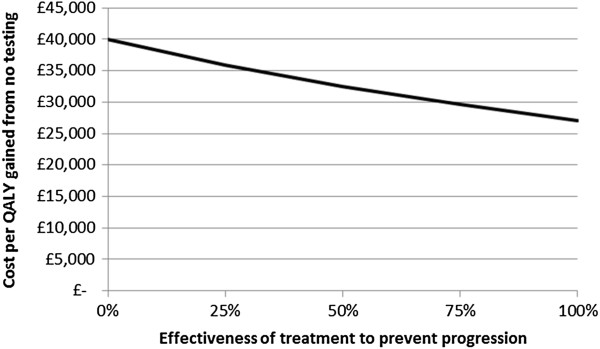
**Sensitivity analysis for the cost per QALY gained for baseline testing, varying chlamydia treatment effectiveness.** Baseline testing strategy is 16.8% overall testing coverage and 0.4 partner notification efficacy. The cost per QALY gained is compared to no testing.

The cost per QALY gained is sensitive to the assumed testing cost (Figure 4) and QALYs gained by testing (Figure 5). The current chlamydia testing strategy in Scotland could be cost-effective if testing is cheaper (the exact cost of testing and treatment in Scotland across all types of settings is unknown), or if morbidity due to PID and TFI is greater, than we estimated. For example, the estimated cost per QALY of 16.8% overall testing coverage compared to no testing would reach threshold cost-effectiveness if testing costs were 20% cheaper (Figure 4), or if the number of QALYs gained was around 30% higher than assumed (Figure 5). We conservatively assumed that treatment does not reduce the risk of progression to PID or TFI, but if treating chlamydia decreases the likelihood of subsequent progression then the cost-effectiveness would also increase (Figure 6). Here, threshold cost-effectiveness would be reached if treatment was 75% effective against progression (i.e., if treatment of chlamydia positives reduced their risk of progression by 75%).

## Discussion

At current (16.8%) overall testing coverage and 0.4 partner notification efficacy, the total annual cost of testing 15–24 year olds for chlamydia in Scotland is around £5.4 million. In the model, increasing testing coverage by 50% from baseline (16.8% overall testing coverage and 0.4 partner notification efficacy) to 25.2% results in *fewer* chlamydia-associated outcomes per year (703 fewer PID, 88 fewer TFI: a 21% decrease in the number of cases occurring). If the overall testing coverage is reduced from current levels by 50% to 8.4%, there will be an *increase* in outcomes (669 additional PID, 84 additional TFI: a 20% increase). Increasing testing coverage generally leads to an increase in the expenditure per case (PID or TFI) averted because proactive testing finds proportionately fewer infections than testing those seeking treatment. However, as partner notification is key to chlamydia control efforts given the high risk of infection within partnerships, increasing partner notification reduces the expenditure per case averted.

Our estimate of the cost per QALY gained of current testing activities is £40,034 under mid-range utility estimates for PID and TFI only, which is above the £20,000-£30,000 threshold for cost-effectiveness used by NICE. The cost-effectiveness of chlamydia testing could be underestimated if: (1) the health state utility value of either PID or TFI applies for longer; (2) treatment costs for the outcomes are higher; (3) ectopic pregnancy and male outcomes are included; and (4) testing coverage is higher among women relative to men. Cost-effectiveness could be overestimated if chlamydia causes fewer outcomes or if chlamydia prevalence is lower. We explored the sensitivity of the cost per QALY gained for some of these in our model and found that cost-effectiveness is highly sensitive to the assumptions made. In general we have made conservative assumptions that would tend to under-estimate cost-effectiveness, meaning testing is likely to be more cost-effective than estimated here.

Our analysis shows that testing at 8.4% coverage is at threshold cost-effectiveness: £28,851 per QALY gain for PID and TFI compared to no testing. However, at lower levels of testing, more chlamydia-associated outcomes will occur and chlamydia prevalence could increase. In the model, treatment seeking individuals would still theoretically get tested if testing coverage was reduced. However, in practice, a reduced focus on testing could reduce chlamydia awareness and therefore reduce the likelihood of individuals seeking treatment, in addition to missing those asymptomatic individuals who are at high risk of having an undiagnosed chlamydia infection, but who are not “treatment seeking” per se. Alternatively, increasing partner notification alone could increase the cost-effectiveness of current chlamydia control (compared to no testing) to NICE threshold levels.

### Limitations

We used a simple deterministic model for analytical tractability while capturing the essential features of the dynamics of chlamydia infection and its complications. Additional model complexity would have required further parameterisation and model fitting which is currently not adequately supported by data and could potentially have hampered model interpretation. However by definition this simple model does not capture all possible factors which could influence the apparent cost-effectiveness of chlamydia testing. We did not explicitly model partnerships, and recovered individuals return to the susceptible pool and experience the same risk of infection as those never infected. The rate of chlamydia reinfection in young women is high [37, 38], which will both reduce the impact of testing on prevalence, and increase the importance of partner notification in controlling the continued spread of infection.

The risk of progression is applied uniformly to all cases of chlamydia at the point of infection acquisition in the absence of reliable estimates of risk of progression to outcomes with duration of infection. We showed in a sensitivity analysis that if we only consider outcomes averted in people never infected, rather than additionally accounting for outcomes averted due to earlier diagnosis and treatment, the effectiveness of testing and treatment to prevent outcomes is underestimated. In addition, the description of progression to PID or TFI from incident chlamydia is one of a number of possible model structures and other structures may predict different effects of treatment [39]. We attempted to overcome this through a sensitivity analysis of progression probabilities.

If testing is done regularly and at high coverage, the mean duration of infection will decrease but potentially reinfections will be more common. We did not incorporate number of infections as a cofactor in progression risks. There is a potential for harm if the risk of secondary outcomes increases with repeat infections [40]; however this has not been observed in countries with high rates of chlamydia testing.

The model used is a deterministic compartmental model which responds very rapidly to changes in the transmission dynamics as the whole population receives the intervention equally and simultaneously. This may overestimate the effectiveness of testing, since in practice the delays and variability inherent in implementing an intervention in a heterogeneous population will slow and reduce its impact.

Lack of data hampers our ability to reliably estimate the level at which partner notification is already done in Scotland, and hence the extent to which this can be increased. Data from England suggest that partner notification efficacy is likely to vary substantially between areas and healthcare settings (NCSP data [29]). Partner notification is a good use of resources, both to treat partners who are highly likely to be infected, and to prevent reinfection in the index case [31]. There is also uncertainty in estimates of partner positivity rates. Theoretical studies have shown that positivity in partners is insensitive to the population prevalence at around 33% [25]. The high positivity observed in men in Scotland suggests that a high number are partners of women with diagnosed chlamydia which would fit with current testing strategy.

The costs of chlamydia testing and treatment need to be better defined for Scotland in order to reliably inform cost-effectiveness calculations, which we showed are sensitive to the assumed costs. For example, nurse-led testing is less expensive than clinician-led testing. More generally, better understanding of the current organisation and provision of chlamydia control activities in different settings in Scotland would help to define how testing guidelines are currently implemented in practice, and thus, what changes could be made to improve the management of chlamydia in the population.

Uncertainty in the amount of morbidity caused by PID and TFI and the associated healthcare costs further reduces the reliability of cost-effectiveness calculations. We used conservative values for the cost of managing a single episode of PID and one round of IVF treatment for TFI. If these costs are higher, for example due to multiple IVF rounds or PID episodes, then our analysis will underestimate the potential cost savings from preventing PID and TFI. A recent systematic review [22] found that there are significant problems in using current available values for health state utility (from which QALYs are derived) for PID and TFI, and in fact in assigning values at all to TFI in particular. Since cost-effectiveness is sensitive to, and indeed dependent on, the assumed health state disutility associated with the outcomes, caution is advised in interpreting the calculated values for the cost per QALY gained for current and alternative chlamydia testing strategies.

## Conclusions

Estimates of the impact and cost-effectiveness of chlamydia testing and treatment to prevent PID and TFI in Scotland are important for guiding local decision-making on the best use of available resources for chlamydia testing and treatment, in order to effectively manage and prevent this common sexually transmitted infection and its serious outcomes. Our findings are based on prevalence, coverage and costs specific to Scotland, but have broader interest to other settings with similar chlamydia management strategies or which are considering a change to their current testing policies. In particular, our finding that partner notification is an efficient use of resources is likely to hold true for other settings [31, 41].

We found that, under current assumptions, current chlamydia testing strategy among young people in Scotland is not cost-effective. However, there is significant potential for current testing strategy to reach threshold cost-effectiveness, as lack of data to inform the calculations obligated the use of conservative assumptions. Significant data gaps exist and the cost-effectiveness calculations should be updated when better parameter estimates, particularly for the health state disutility associated with PID and TFI, become available. Although lower testing coverage is at threshold cost-effectiveness, we would expect to see more PID and TFI cases with less testing. Scotland has high chlamydia test positivity even at 16.8% coverage, and decreasing testing could lead to an increase in chlamydia prevalence, and uncertain future burden of adverse reproductive morbidities.

Until cost-effectiveness calculations can be more reliably informed, particularly with regard to the health state utilities, and on the basis of current model and parameter uncertainty, maintaining current coverage, but improving the efficiency of the testing and treatment process would be advisable. One such way would be to increase the extent to which partner notification is done, which is cost-effective for any increase in partner notification level compared to the current testing strategy.

## Electronic supplementary material

Additional file 1: **Impact and cost-effectiveness of chlamydia testing in Scotland: a mathematical modelling study - supplementary model details and modelling results** [42] **.** (PDF 1 MB)
