# Supplementary material for: Impact and cost-effectiveness of chlamydia testing in Scotland: a mathematical modelling study
Source: Theor Biol Med Model. 2015 Jan 15;12:2. doi: 10.1186/1742-4682-12-2 (PMC4429484; doi:10.1186/1742-4682-12-2)
Supplement: Supplementary file 1 — Additional file 1: Impact and cost-effectiveness of chlamydia testing in Scotland: a mathematical modelling study - supplementary model details and modelling results [42] . (PDF 1 MB) [file 12976_2014_504_MOESM1_ESM.pdf]

ADDITIONAL FILE 1

Impact and cost-effectiveness of chlamydia testing in Scotland: a mathematical modelling study – supplementary model details and modelling results

Authors: Looker, K. J., Wallace, L. A. and Turner, K. M. E.

Model equations

The model equations are given by:

$$\frac{d}{dt}S_i(t) = N * \varphi * r_i - \lambda_i(t) * S_i(t) - \alpha * S_i(t)$$

$$\frac{d}{dt}CT_i(t) = \lambda_i(t) * S_i(t) - (COV * CT_i * D_{TREATED}) - (COV * CT_i * D_{TREATED} * PNe * POS_{PN}) - (SEEKTREAT_{INF} * CT_i * D_{TREATED}) - (SEEKTREAT_{INF} * CT_i * D_{TREATED} * PNe * POS_{PN}) - \delta * CT_i - \alpha * CT_i(t)$$

$$\frac{d}{dt}R_i(t) = -\lambda_i(t) * R_i(t) + (COV * (CT_i + CT'_i) * D_{TREATED}) + (COV * (CT_i + CT'_i) * D_{TREATED} * PNe * POS_{PN}) + (SEEKTREAT_{INF} * (CT_i + CT'_i) * D_{TREATED}) + (SEEKTREAT_{INF} * (CT_i + CT'_i) * D_{TREATED} * PNe * POS_{PN}) + \delta * (CT_i + CT'_i) - \alpha * R_i(t)$$

$$\frac{d}{dt}CT'_i(t) = \lambda_i(t) * R_i(t) - (COV * CT'_i * D_{TREATED}) - (COV * CT'_i * D_{TREATED} * PNe * POS_{PN}) - (SEEKTREAT_{INF} * CT'_i * D_{TREATED}) - (SEEKTREAT_{INF} * CT'_i * D_{TREATED} * PNe * POS_{PN}) - \delta * CT'_i - \alpha * CT'_i(t)$$

where, at time *t*, N is the total population size, $S_i(t)$ is the number of individuals in sexual activity class *i* susceptible to chlamydia, $CT_i(t)$ is the number of individuals in sexual activity class *i* infected with chlamydia for the first time, $R_i(t)$ is the number of recovered individuals in sexual activity class *i*, $CT'_i(t)$ is the number of individuals in sexual activity class *i* reinfected with chlamydia, φ is the rate of entry (ageing) into the population, r_i is the proportion of individuals recruited into sexual activity class *i* on entry into the model, α is the rate of exit (ageing) from the population, and $\lambda_i(t)$ is the force of infection per susceptible (or recovered) in sexual activity class *i*. The force of infection is calculated as follows:

$$\lambda_i(t) = \beta * c_i * \sum_j \left[\rho_{ij} * \frac{(CT_j + CT'_j)(t)}{N_j}\right]$$

where β is the transmission probability per partnership, c_i is the partner change rate per year for individuals in sexual activity class *i*, $\frac{(CT_j+CT'_j)(t)}{N_j}$ is the chlamydia prevalence among partners in sexual activity class *j*, and the ρ_{ij} is the proportion of partnerships of individuals in sexual activity class *i* formed with individuals in sexual activity class *j*. Here ρ_{ij} is given by:

$$\rho_{ij} = \varepsilon * \delta_{ij} + (1 - \varepsilon) * \left(\frac{c_j * N_j}{\sum_j c_j * N_j}\right)$$

where δ_{ij}=1 for *i=j* and 0 otherwise[42] and ε is the proportion of partnerships that are formed with individuals in the same sexual activity class over and above the proportion that would occur through random mixing.

$SEEKTREAT_{INF}$ is the proportion of infected individuals who seek treatment, *COV* is the proportion of individuals who are additionally tested, and $D_{TREATED}$ is the proportion of individuals identified as positive (treatment seeking or additional testing) who are successfully treated. Natural recovery from infection is denoted by δ. The effect of partner notification is incorporated in the model by moving additional infected individuals ($PNe * POS_{PN}$) into the recovered class per treated positive (treatment seeking or additional testing), where *PNe* is the number of partners notified and tested/treated per (treated) positive index, and POS_{PN} is the chlamydia positivity among partners.

For females, the risk of PID per incident chlamydia infection was taken to be 0.16 (16%)[17], while the risk of TFI per incident chlamydia infection was taken to be 0.02 (2%)[27].

Table S1 Model outputs.

All outputs are applied to the Scottish population.

Output	Symbol	Equation
Prevalence	*PREV*	= (ARRAYSUM(CT[*]) + ARRAYSUM(CT'[*])) / 10000 * 100
Number of additional tests	*Total_additional_tests*	= COV * (N_{TARGET_F} + N_{TARGET_F})
Number of partner notification tests	*Total_PN_tests*	= (((PNe * COV * ARRAYSUM(CT[*]) * $D_{TREATED}$) + (PNe * COV * ARRAYSUM(CT'[*]) * $D_{TREATED}$) + (PNe * $SEEKTREAT_{INF}$ * ARRAYSUM(CT[*]) * $D_{TREATED}$) + (PNe * $SEEKTREAT_{INF}$ * ARRAYSUM(CT'[*]) * $D_{TREATED}$)) / 10000) * (N_{TARGET_F} + N_{TARGET_F})
Number of treatment seeking tests	*Total_TS_tests*	((($SEEKTREAT_{INF}$ * ARRAYSUM(CT[*])) / $POS_{SEEKTREAT}$) + (($SEEKTREAT_{INF}$ * ARRAYSUM(CT'[*]) / $POS_{SEEKTREAT}$))) / 10000) * (N_{TARGET_F} + N_{TARGET_F})
Total number of tests	*Total_tests*	= Total_additional_tests + Total_PN_tests + Total_TS_tests
Number identified as being infected and successfully treated	*$INF_{TREATED}$*	= (Total_PN_tests * POS_{PN}) + (Total_TS_tests * $POS_{SEEKTREAT}$ * $D_{TREATED}$) + (((COV * (ARRAYSUM(CT[*]) + ARRAYSUM(CT'[*])) * $D_{TREATED}$) / 10000) * (N_{TARGET_F} + N_{TARGET_F}))
Total cost of testing and PN, £	*Total_cost*	= (Total_additional_tests * C_{TEST}) + (Total_PN_tests* C_{PN}) + (Total_TS_tests * C_{TEST})
Cost per infected individual successfully identified and treated, £	*$COST_{PER\_TREATED}$*	= Total_cost / $INF_{TREATED}$
Rate of PID per year per 1,000 females	*PID_1000*	= ((PID_risk * Incidence / 2) / 5000) * 1000
Rate of TFI per year per 1,000 females	*TFI_1000*	= ((TFI_risk * Incidence / 2) / 5000) * 1000
Rate of PID per year per 1,000 females at baseline	*Baseline_PID_1000*	= ((PID_risk * Incidence / 2) / 5000) * 1000; TEST = 16.8%
Rate of TFI per year per 1,000 females at baseline	*Baseline_TFI_1000*	= ((TFI_risk * Incidence / 2) / 5000) * 1000; TEST = 16.8%
Rate of PID per year per 1,000 females with no chlamydia testing	*PID_1000_with_no_CT_testing*	= ((PID_risk * Incidence / 2) / 5000) * 1000; TEST = 0%, $SEEKTREAT_{INF}$ = 0
Rate of TFI per year per 1,000 females with no chlamydia testing	*TFI_1000_with_no_CT_testing*	= ((TFI_risk * Incidence / 2) / 5000) * 1000; TEST = 0%, $SEEKTREAT_{INF}$ = 0
Number of PID cases prevented per year from a baseline overall testing coverage of 16.8%	*PID_prevented*	= ((Baseline_PID_1000 - PID_1000) / 1000) * N_{TARGET_F}
Number of TFI cases prevented per year from a baseline overall testing coverage of 16.8%	*TFI_prevented*	= ((Baseline_TFI_1000 - TFI_1000) / 1000) * N_{TARGET_F}
Expenditure on testing and treatment per PID case prevented from no chlamydia testing or partner notification, £	*$COST_{PER\_PID\_PREVENTED}$*	= Total_cost / (((PID_per_1000F_with_no_CT_testing - PID_1000) / 1000) * N_{TARGET_F})
Expenditure on testing and treatment per TFI case prevented from no chlamydia testing or partner notification, £	*$COST_{PER\_TFI\_PREVENTED}$*	= Total_cost / (((TFI_per_1000F_with_no_CT_testing - TFI_1000) / 1000) * N_{TARGET_F})
Total number identified as chlamydia positive	*INF_{TESTED}*	= (Total_PN_tests * POS_{PN}) + (Total_TS_tests * $POS_{SEEKTREAT}$) + (((COV * (ARRAYSUM(CT[*]) + ARRAYSUM(CT'[*]))) / 10000) * (N_{TARGET_F} + N_{TARGET_F}))
Test positivity	*Test_positivity*	= INF_{TESTED} / Total_tests

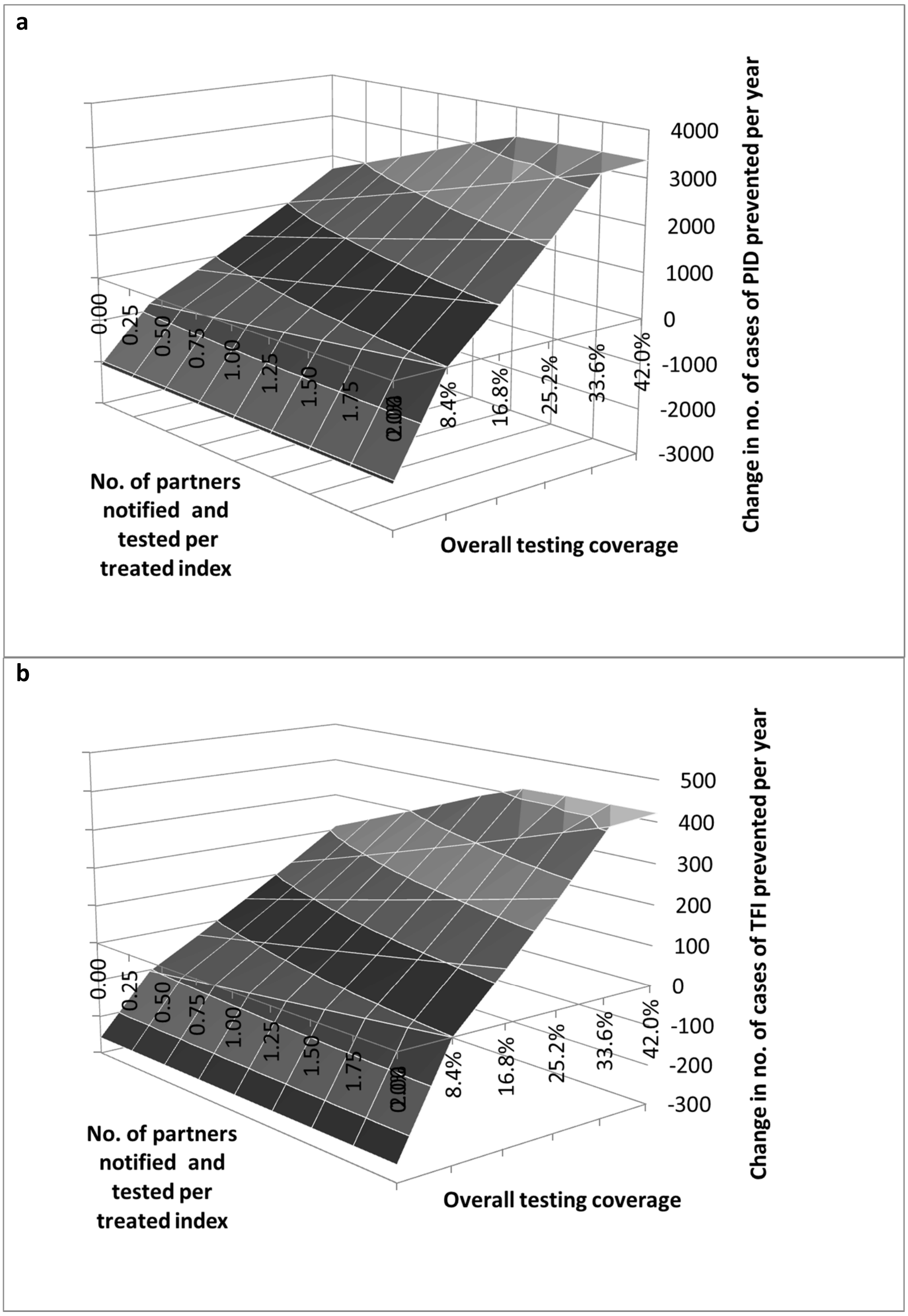

Figure S1 Change in the number of (a) PID and (b) TFI cases prevented annually as a function of testing strategy.

Numbers of cases prevented are among 15-24 year old women in Scotland compared to current chlamydia testing strategy (16.8% overall testing coverage and 0.4 partner notification efficacy) and are for chlamydia-attributable cases only. The greyscale represents the area between the gridlines on the vertical (y) axis. Calculations are for equilibrium state.

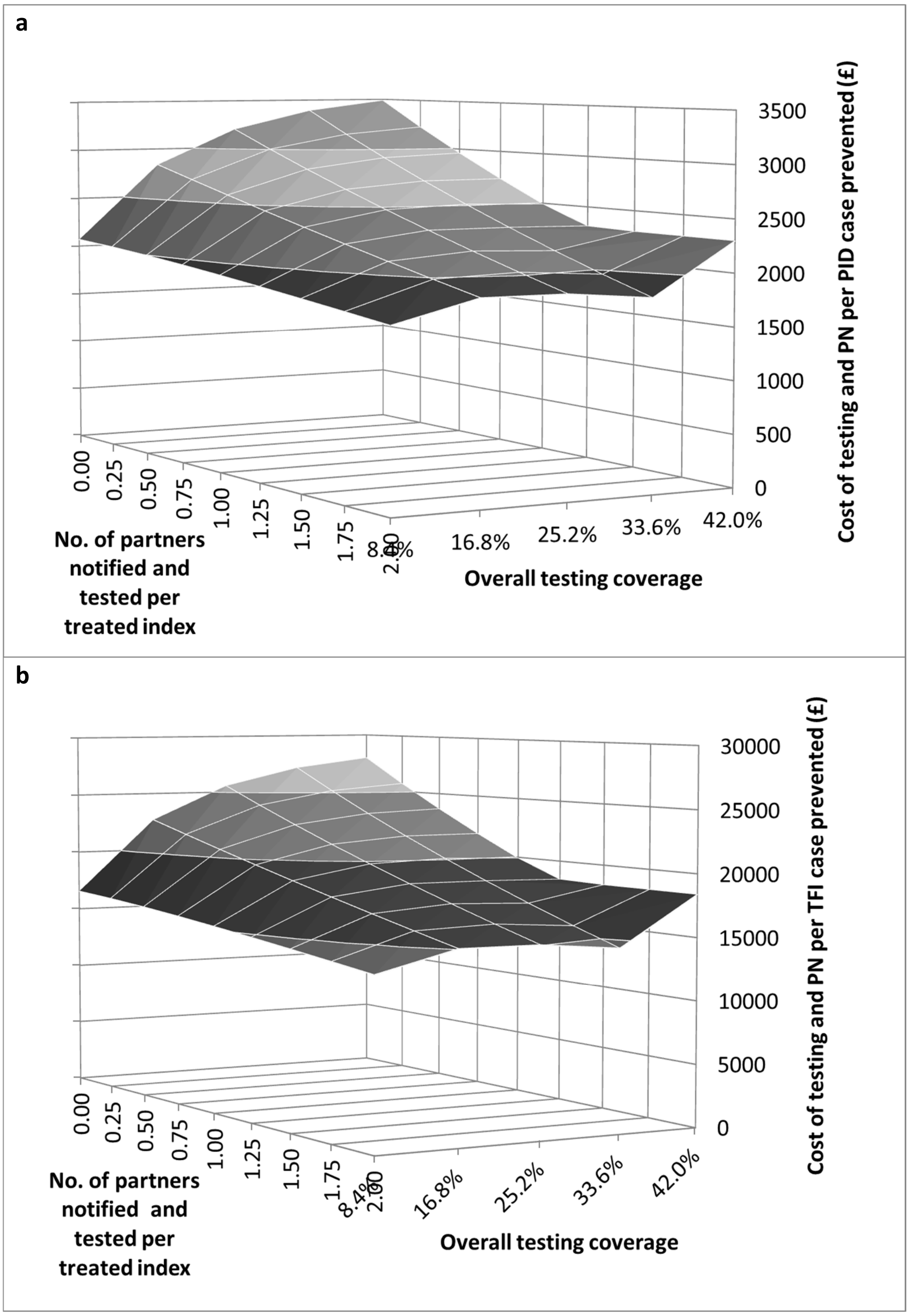

Figure S2 Expenditure on testing per (a) PID and (b) TFI case prevented as a function of testing strategy.

Expenditure on testing and numbers of cases prevented are for 15-24 year old women in Scotland compared to no testing. Cases prevented are for chlamydia-attributable cases only. The greyscale represents the area between the gridlines on the vertical (y) axis. This figure does not incorporate the cost saving of PID/TFI treatment for averted cases. Calculations are for equilibrium state.

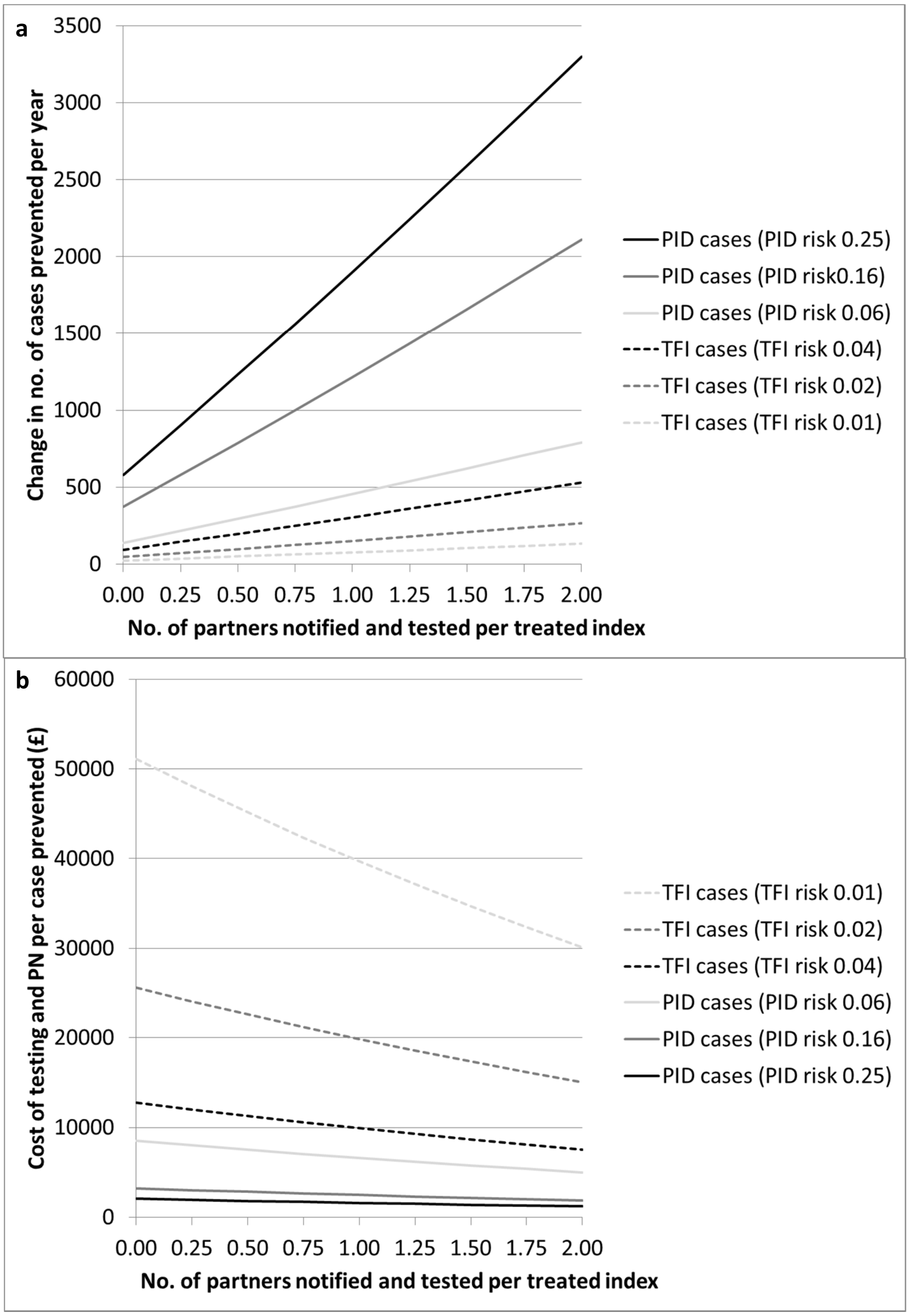

Figure S3 Sensitivity analysis for (a) the change in the number of PID/TFI cases prevented and (b) the expenditure on testing and treatment per PID/TFI case prevented, as a function of PID/TFI risk.

Numbers of cases prevented are among 15-24 year old women in Scotland compared to current chlamydia testing strategy (16.8% overall testing coverage and 0.4 partner notification efficacy) and are for chlamydia-attributable cases only. Expenditure on testing and numbers of cases prevented are for 15-24 year old women in Scotland compared to no testing. Cases prevented are for chlamydia-attributable cases only. This figure does not incorporate the cost saving of PID/TFI treatment for averted cases. In these figures overall testing coverage is set at 25.2%.

Table S2 (To accompany Figure 2.) Cost per QALY gained for different levels of overall testing coverage compared to no testing. Partner notification efficacy is 0.4. Note that there is no valid cost per QALY gained for no testing. Calculations are for equilibrium state. Also shown is the incremental cost per QALY gained in moving from one level of coverage to the next.

	**Overall testing coverage**					
	**0.0%**	**8.4%**	**16.8%**	**25.2%**	**33.6%**	**42.0%**
Total cost of chlamydia testing (£millions)	0	2.81	5.42	8.00	10.55	13.07
No. PID cases averted from no testing	0	1393	2062	2765	3503	4277
No. TFI cases averted from no testing	0	174	258	346	438	535
Health state utility value (PID) (applies for 3 months)	0.9	0.9	0.9	0.9	0.9	0.9
Health state utility value (TFI) (applies for 1 year)	0.76	0.76	0.76	0.76	0.76	0.76
QALYs lost per PID case (adjusted)	0.025	0.025	0.025	0.025	0.025	0.025
QALYs lost per TFI case (adjusted)	0.24	0.24	0.24	0.24	0.24	0.24
Difference in testing cost (£millions)	0	2.81	5.42	8.00	10.55	13.07
QALYs gained by new scenario	0	77	113	152	193	235
Cost saving due to costs of outcomes averted (£)	--	595,424	881,268	1,181,732	1,497,034	1,827,845
**Cost per QALY gained (compared to no testing)**	**--**	**£ 28,851**	**£ 40,034**	**£ 44,836**	**£ 46,983**	**£ 47,806**
Incremental cost per QALY gained (moving from one coverage level to the next)	--	£ 28,851 (0.0% to 8.4%)	£ 63,329 (8.4% to 16.8%)	£ 58,921 (16.8% to 25.2%)	£ 55,027 (25.2% to 33.6%)	£ 51,530 (33.6% to 42.0%)

Table S3 (To accompany Figure 3.) Incremental cost per QALY gained if partner notification efficacy is changed from baseline (0.4). Overall testing coverage is 16.8%. Note that there is no valid cost per QALY gained for the baseline strategy. Calculations are for equilibrium state. Also shown is the overall cost per QALY gained for each scenario compared to no testing. *Net monetary saving compared to baseline, but more outcomes occur (more QALYs are lost).

	**Number of partners notified and tested/treated per treated index**									
	**0**	**0.25**	**0.4**	**0.5**	**0.75**	**1**	**1.25**	**1.5**	**1.75**	**2**
Total cost of chlamydia testing (£millions)	5.16	5.33	5.42	5.48	5.59	5.69	5.75	5.79	5.81	5.80
No. PID cases averted from baseline	-242	-91	0	61	216	372	531	691	853	1017
No. TFI cases averted from baseline	-30	-11	0	8	27	47	66	86	107	127
Health state utility value (PID) (applies for 3 months)	0.9	0.9	0.9	0.9	0.9	0.9	0.9	0.9	0.9	0.9
Health state utility value (TFI) (applies for 1 year)	0.76	0.76	0.76	0.76	0.76	0.76	0.76	0.76	0.76	0.76
QALYs lost per PID case (adjusted)	0.025	0.025	0.025	0.025	0.025	0.025	0.025	0.025	0.025	0.025
QALYs lost per TFI case (adjusted)	0.24	0.24	0.24	0.24	0.24	0.24	0.24	0.24	0.24	0.24
Difference in testing cost (£)	-264,910	-90,740	0	54,900	172,860	263,980	329,020	368,700	383,720	374,710
QALYs gained by new scenario	-13	-5	0	3	12	20	29	38	47	56
Cost saving due to costs of outcomes averted (£)	-103,475	-39,049	0	26,196	92,259	159,137	226,828	295,331	364,646	434,770
**Incremental cost per QALY gained**	**£ 12,123***	**£ 10,286***	**-- (Baseline)**	**£ 8,515**	**£ 6,789**	**£ 5,119**	**£ 3,501**	**£ 1,930**	**£ 406**	**£ -1,073**
Cost per QALY gained (compared to no testing)	£ 43,747	£ 41,414	£ 40,034	£ 39,124	£ 36,884	£ 34,694	£ 32,556	£ 30,470	£ 28,436	£ 26,454
